# The Christmas Delineator

**Published:** 1904-12

**Authors:** 


**THE CHRISTMAS DELINEATOR.**

The December *Delineator*, with its message of good cheer and helpfulness, will be welcomed in every home. The fashion pages are unusually attractive, illustrating and describing the very latest modes in a way to make their construction during the busy festive season a pleasure instead of a task, and the literary and pictorial features are of rare excellence. A selection of Love Songs from the Wagner Operas, rendered into English by Richard de Gallienne and beautifully illustrated in colors by J. C. Leyendecker, occupies a prominent place, and a chapter in the Composers' Series, relating the Romance of Wagner and Cosima, is an interesting supplement to the lyrics. A very clever paper entitled "The Court Circles of the Republic," describes some unique phases of Washington social life is from an unnamed contributor, who is said to write from the inner circles of society. There are short stories from the pens of F. Hopkinson Smith, Robert Grant, Alice Brown, Mary Stewart Cutting and Elmore Elliott Peake, and such interesting writers as Julia Magruder, L. Frank Baum, and Grace MacGowan Cooke hold the attention of the children. Many Christmas suggestions are given in needlework and the Cookery pages are redolent of the Christmas feast. In addition, there are the regular departments of the magazine, with many special articles on topics relating to woman's interests within and without the home.

